# Treatment delay from aneurysmal subarachnoid hemorrhage to endovascular treatment: a high-volume hospital experience

**DOI:** 10.1186/s41016-021-00262-0

**Published:** 2021-10-05

**Authors:** Xiaoxi Zhang, Haishuang Tang, Qiao Zuo, Gaici Xue, Guoli Duan, Yi Xu, Bo Hong, Rui Zhao, Pengfei Yang, Jianmin Liu, Qinghai Huang

**Affiliations:** 1 Department of Neurosurgery, Changhai Hospital, Naval Military Medical University, 168 Changhai Rd, Shanghai, 200433 People’s Republic of China; 2 Naval Medical Center of PLA, Naval Military Medical University, Shanghai, 200050 People’s Republic of China

**Keywords:** Intracranial aneurysm, Rupture, Coiling with stent placement, Procedure-related complication, Safety

## Abstract

**Background:**

Early treatment for patients with aneurysmal subarachnoid hemorrhage (aSAH) could significantly reduce the risk of re-bleeding and improve clinical outcomes. We assessed the different time intervals from the initial hemorrhage, admission, and endovascular treatment and identified the risk factors contributing to delay.

**Methods:**

Between February 2017 and December 2019, 422 consecutive aSAH patients treated in a high-volume hospital were collected and reviewed. Risk factors contributing to admission delay and treatment delay were analyzed with univariate and multivariate analysis.

**Results:**

One hundred twenty-two (28.9%) were admitted to the high-volume hospital at the day of symptom onset and 386 (91.5%) were treated with endovascular management at the same day of admission. The multivariate analysis found that younger age (*P* = 0.022, OR = 0.981, 95% CI 0.964–0.997) and good Fisher score (*P* = 0.002, OR = 0.420, 95% CI 0.245–0.721) were independent risk factors of admission delay. None was found to be related with treatment delay. Multivariate analysis (OR (95% CI)) showed that higher age 1.027 (1.004–1.050), poorer Fisher score 3.496 (1.993–6.135), larger aneurysmal size 1.112 (1.017–1.216), and shorter interval between onset to admission 1.845 (1.018–3.344) were independent risk factors of poorer clinical outcome.

**Conclusion:**

Treatment delay was mainly caused by pre-hospital delay including delayed admission and delayed transfer. Our experience showed that cerebrovascular team could provide early treatment for aSAH patients. Younger age and good Fisher score were significantly related with admission delay. However, admission delay was further significantly correlated with better clinical outcome.

## Background

Aneurysmal subarachnoid hemorrhage (aSAH) universally leads to pernicious outcomes if not well managed [1]. The mortality rate of subarachnoid hemorrhage decreased by approximately 50% over the last 20 years, mainly due to improved prognosis in patients surviving to reach hospitals [2]. However, no significant reductions in incidence of SAH and 30-day case fatality were observed in previous studies [3]. The pre-hospital mortality rate of aSAH was 15% [4]. The early re-bleeding rate within 24 h was 5.8%, with a median time interval from initial hemorrhage to re-bleeding of 180 min universally leading to worse clinical outcome [5, 6]. The incidence of re-bleeding progressively also increased with longer interval between initial hemorrhage and treatment [7]. Thereafter, early treatment of responsible ruptured aneurysms was the basic principles in managing aSAH patients. Early treatment could significantly reduce re-bleeding rate and improve clinical outcomes, especially in poor-grade patients [8–10]. Menno et al. reported the time interval between initial hemorrhage and treatment, with 76% patients treated by coiling or clipping within 24 h after diagnosis [11]. Sarmiento et al. found that clipping was an independent risk factor of treatment delay [12]. Here, we reported the time intervals between initial hemorrhage, admission, and endovascular treatment and related risk factors and our time principles in managing aSAH patients.

## Methods

### Patient selection and population

Institutional review board-approved protocol was obtained prior to study enrollment. Consecutive aSAH patients treated with endovascular regimens between January 2017 and December 2019 in our hospital were collected. Time points were retrospectively retrieved from the medical record system and were reviewed independently by two authors. The angiographic and clinical data were reviewed by three experienced endovascular surgeons. Written informed consent was obtained from all patients.

Inclusion criteria for this study were acutely ruptured aneurysm, defined as aneurysms treated within 28 days since initial hemorrhage. Exclusion criteria were (a) fusiform, traumatic, blood blister-like, tumor-related, and AVM-related aneurysms. From January 2017 to December 2019, 641 patients with ruptured intracranial aneurysms were treated in our hospital. According to the patient selection criteria, 422 consecutive aSAH patients were included in this study.

### Procedure technique and medications

Patients were all treated by five endovascular neurosurgeons, all with more than 15 years of experience. All procedures were performed under general anesthesia with a trans-femoral approach and systemic heparinization, aiming at maintaining an activated clotting time of 2 to 3 times baseline.

A 6-F guiding catheter was delivered into the distal part of internal carotid artery or vertebral artery. All materials including microcatheters, stents, and coils were delivered through this catheter. The stents and coils were deployed according to standard procedure, as recommended by the manufacturer. If acute thrombosis occurred during the procedure, glycoprotein IIb/IIIa inhibitor (Tirofiban; Grand Pharma, Wuhan, China) was used.

A loading dose of aspirin and clopidogrel (300 mg each) was administered orally or rectally intra-operatively after a decision was made to perform coiling with stent placement. In the postoperative period, patients who underwent coiling with stent placement were maintained on a dose of 100 mg aspirin and 75 mg clopidogrel daily for 6 weeks, followed by 100 mg aspirin daily alone which was continued indefinitely. Antiplatelet therapy was not given in any of the patients who received coiling without stent either intra-operatively or post-operatively.

### Clinical and angiographic evaluation

Clinical outcomes were evaluated at discharge based on the modified Rankin Scale (mRS). Favorable outcomes were defined as a mRS score of 0 to 2 and poor outcomes were defined as a mRS score of 3 to 6. Angiographic results obtained with digital subtraction angiography were categorized into three categories: (1) complete occlusion, with no contrast medium observed on DSA; (2) neck remnant, persistence of any portion of the original defect in the arterial wall; and (3) incomplete occlusion, any opacification of the aneurysmal sac. The same two physicians who evaluated the immediate angiographic results also evaluated the angiographic follow-up studies.

### Statistical analysis

Time intervals were calculated based on medical records system in our hospital. The Fisher score and Hunt-Hess score were dichotomized into groups with a good Fisher (0~2) or Hunt-Hess score (I~III) and with a poor Fisher (3~4) or Hunt-Hess score (IV~V). Admission delay was considered as admission to our hospital at the third day of initial hemorrhage or later. Treatment delay was considered as treatment at the second day after admission to our hospital or later.

Normally distributed variables were expressed as mean ± standard deviation (SD) and tested with Student’s *t* test (two group comparison). Unequally distributed variables were expressed as medians with interquartile ranges and tested with Mann-Whitney *U* test. Categorical variables were expressed as frequency and tested with Pearson *χ*^2^ test. Potential risk factors related to treatment delay (patient age and sex, Hunt-Hess score, Fisher score, aneurysm size, admission time) were categorized and analyzed in Table 1 and Table 2. Univariate analysis was performed to determine the correlation between risk factors and treatment delay. Statistical significance was considered to be 0.05. Statistical analysis was performed by using SPSS version 22.0 software.

**Table 1:** Baseline characteristics of 422 patients with aSAH

	Aneurysms (%)
Sex (male/female)	147/275
Age (year)	56.81 ± 12.00
Hunt-Hess Score	
I	200 (47.4)
II	111 (26.3)
III	68 (16.1)
IV	40 (9.5)
V	2 (0.5)
Fisher Score	
0	2 (0.5)
1	11 (2.6)
2	324 (76.8)
3	83 (19.7)
4	4 (0.9)
Aneurysmal Size (mm)	5.15 ± 2.77
Location	
PcomA	138 (32.7)
AcomA	135 (32.0)
MCA	54 (12.8)
ICA	39 (9.2)
PC	29 (6.9)
ACA	27 (6.4)

PcomA, posterior communicating artery; AcomA, anterior communicating artery; MCA, middle cerebral artery; ICA, internal cerebral artery; PC, posterior circulation; ACA, anterior cerebral artery

**Table 2:** Time intervals between onset, admission, and endovascular treatment

	Onset-admission	Onset-treatment	Admission-treatment
The 1st day	122 (28.9%)	117 (27.7%)	386 (91.5%)
2~3 days	209 (49.5%)	214 (50.7%)	32 (7.6%)
4~14 days	79 (18.7%)	77 (18.3%)	4 (1.0%)
15 days	12 (2.8%)	14 (3.3%)	0

## Results

### Patient characteristics

The mean age of included patients were 56.81 ± 12.00 years (range 24~88 years) and 275 patients (65.2%) were female. Good Hunt-Hess score (I~III) and Fisher score (0~2) was obtained in 89.8% and 79.4%, respectively. Most patients presented with Hunt-Hess score of I~II (73.3%) and Fisher score of 2 (76.8%). Previous hypertension was observed in 195 patients (46.2%), diabetes in 29 patients (6.9%) and cerebrovascular disease other than aSAH in 28 patients (6.6%). Among the included aneurysms, 32.7% were located on posterior communicating artery.

### Time intervals

Only 28.9% patients (*n* = 122) were admitted to our hospital at the day of onset. 78.5% patients (*n* = 331) were admitted within 3 days. Twelve patients (2.8%) were admitted after 14 days. Three hundred thirty-one patients (78.4%) were treated within 3 days after onset. Three hundred eighty-six patients (91.5%) were treated at the first day of admission to our hospital, as shown on Table 2.

### Predictors of delay

As shown on Table 3, univariate analysis showed that younger age (*P* = 0.018) and good Fisher score (*P* = 0.001) were significantly related with admission delay. None was observed to be related with treatment delay. Admission delay was also significantly related with treatment delay (*P* < 0.001). The multivariate analysis found that younger age (*P* = 0.022, OR = 0.981, 95% CI 0.964–0.997) and good Fisher score (*P* = 0.002, OR = 0.420, 95% CI 0.245–0.721) were independent risk factors of admission delay.

**Table 3:** Univariate analysis of the correlation between admission delay and treatment delay

	Onset-admission				Admission-treatment		
	In time	Delayed	*P*		In time	Delayed	*P*
Sex			0.404				0.579
Male	85 (57.8)	62 (42.2)			133 (90.5)	14 (9.5)	
Female	171 (62.2)	104 (37.8)			254 (92.4)	21 (7.6)	
Age	57.9 ± 11.6	55.1 ± 12.5	0.018		57.0 ± 12.0	55.2 ± 12.4	0.410
Hypertension			0.618				0.033
Yes	121 (62.1)	74 (37.9)			185 (94.9)	10 (5.1)	
No	135 (59.5)	92 (40.5)			202 (89.0)	25 (11.0)	
CHD			0.417				0.187
Yes	7 (50.0)	7 (50.0)			11 (78.6)	3 (21.4)	
No	249 (61.0)	159 (39.0)			376 (92.2%)	32 (7.8)	
Diabetes			0.432				0.528
Yes	20 (69.0)	9 (31.0)			28 (96.9)	1 (3.4)	
No	236 (60.1)	157 (39.9)			359 (91.3)	34 (8.7)	
Cerebrovascular disease history			1.000				1.000
Yes	17 (60.7)	11 (39.3)			26 (92.9)	2 (7.1)	
No	239 (60.7)	155 (39.3)			361 (91.6)	33 (8.4)	
Hunt-Hess Score			0.084				0.388
I~III	45 (52.3)	41 (47.7)			77 (89.5)	9 (10.5)	
IV~V	211 (62.8)	125 (37.2)			310 (92.3)	26 (7.7)	
Fisher Score			0.001				1.000
0~2	190 (56.7)	145 (43.3)			307 (91.6)	28 (8.4)	
3~4	66 (75.9)	21 (24.1)			80 (92.0)	7 (8.0)	
Admission time			0.155				0.847
Weekday	174 (58.4)	124 (41.6)			274 (91.9)	24 (8.1)	
Weekend	82 (66.1)	42 (33.9)			113 (91.1)	11 (8.9)	
Aneurysmal Size	5.24 ± 2.88	5.00 ± 2.60	0.370		5.19 ± 2.84	4.70 ± 1.87	0.319
Onset-admission			–				< 0.001
In time	–	–			252 (98.4)	4 (1.6)	
Delayed	–	–			135 (81.3)	31 (18.7)	

### Delay with clinical outcome

According to the time intervals between onset, admission, and treatment, there was a significant correlation between admission delay with between clinical outcome (23.7% vs. 42.8%) (Table 4).

**Table 4:** Correlation between delay with clinical outcome

	Onset-admission				Admission-treatment		
	In time	Delayed	*P*		In time	Delayed	*P*
mRS at discharge			0.002				0.489
0~2	198 (57.2)	148 (42.8)			319 (92.2)	27 (7.8)	
3~6	58 (76.3)	18 (23.7)			68 (89.5)	8 (10.5)	

### Outcome

Clinical outcome was assessed in all patients at discharge. Seventy-six patients (18.0%) had a poor outcome, with 14 patients (3.3%) died. Higher age, larger aneurysmal size, poorer Fisher score, and shorter interval between onset and admission and shorter interval between onset and treatment were found to be significantly related with poorer clinical outcome. Multivariate analysis (OR (95% CI)) showed that higher age 1.027 (1.004–1.050), poorer Fisher score 3.496 (1.993–6.135), larger aneurysmal size 1.112 (1.017–1.216), and shorter interval between onset to admission 1.845 (1.018-3.344) were independent risk factors of poorer clinical outcome.

## Discussion

In this study, different time intervals from initial hemorrhage to admission and followed by endovascular treatment were reported. Prompt treatment (the same day of admission) after admission to our hospital was observed in over 90% aSAH patients. Unfortunately, the major delay to endovascular treatment was pre-hospital delay including delayed admission and delayed transfer rather than in-hospital delay. Multivariate analysis showed that younger age and good Fisher score were independent risk factors for admission delay.

Over 90% patients were treated at the day of admission, namely, the interval between admission to endovascular treated was less than 24 h in most patients. The interval between admission to endovascular treatment was considerably shorter in our hospital than any other previous studies [13]. Sarmiento et al. reported the time intervals between admission and treatment of 38,827 aSAH patients, with 9.3% patients not treated within 2 days after admission [12]. Potential explanations for treatment delay in aSAH patients are the lack of aneurysm team available for 24/7, lack of management experience due to low volume of aneurysm cases, lack of routine provision of clipping or coiling at weekends and nighttime, etc. In our hospital, four cerebrovascular teams (with over 90% of cerebrovascular disease and 40~60% of intracranial aneurysms) were established, with at least one team on duty for 24/7. The annual number of patients with intracranial aneurysms admitted to our hospital was also increased, with annual over 700 aneurysms since 2016. With nine interventionalists that could handle intracranial aneurysms, we have the ability to treat the intracranial aneurysms 24/7 and initialize endovascular treatment as soon as possible, even in nighttime. In addition, CT, DSA and CTA were all available for 24/7, making it possible to provide full-time and prompt treatment for aSAH patients.

The clinical outcome of the patients in this study was comparable in comparison to large aSAH studies [14, 15]. An explanation for this might be ultra-early treatment for admitted patients. The following policy was applied to treat aSAH patients as soon as possible: (1) the concept of “time is brain” was extrapolated to patients with aSAH. The 24/7 h policy of thrombolysis and thrombectomy for patients with acute ischemic stroke was also extrapolated to aSAH patients. (2) Physicians and interventionalists were prepared when the patients were still in transport. (3) Patients with aSAH were immediately delivered to the operation room to receive endovascular treatment after admission. In addition, the application of pre-hospital and in-hospital logistics for ultra-early treatment of acute ischemic stroke would significantly reduce the delay of admission and treatment [16–18]. CT-unit in the ambulance has been used for patients with acute ischemic stroke in China, which shortens the time to diagnosis and treatment [19]. It might also be used for patients with aSAH.

The treatment of intracranial aneurysms is more concentrated in high-volume hospitals in China. The impact of case volume of hospitals on the management of aSAH patients have been reported in previous studies [20, 21]. High-volume hospitals were related with lower mortality rate and better clinical outcome. Thereafter, aSAH patients were recommended to be transferred to high-volume hospitals if possible. Low-volume hospitals were also recommended to establish cerebrovascular team, with cerebrovascular surgeons and endovascular specialists that could provide multidisciplinary and systematic neuro-intensive care. Since quantitative patients were admitted, a high-volume hospital could provide 24/7 prompt and experienced treatment for aSAH patients. Our experience indicated that cerebrovascular team could significantly improve the medical service for aSAH patients, including ultra-early treatment and better clinical outcome.

Although the intervals between admission to endovascular treatment were short, there is still room for improvement to optimize ultra-early aneurysm treatment. Pre-hospital logistics could be optimized by immediate and direct transfer from primary hospitals to treatment centers or high-volume hospitals. Similar with acute ischemic stroke, “time is brain” principle should be obeyed when transferring aSAH patients.

Restrictions should be highlighted of this study. Firstly, all data were retrieved from medical record system in our hospital. Thus, treatment delay caused by transfer to primary medical centers would be impended. Secondly, this study was a retrospective single-center study and thus could not provide extrapolation of currently overall treatment status of aSAH, since time intervals were different among countries, regions, and even hospitals [22, 23]. Thirdly, the influence of admission delay time discussed in our paper was more focused on patient themselves; the factors of hospital and doctors were less discussed, which may need more attentions in further studies. Lastly, the time points were retrieved from the medical records system of our hospital and precise time could not be retrieved in all patients. Thus, the measurement unit of time intervals would be day rather than hours or minutes, which we considered proper and rational in our hospital. Lastly, emergency treatment policy for aSAH was quite different among hospitals. In our hospital, aSAH was considered to be disastrous which needed to be treated immediately if possible. However, this policy is rarely seen in most hospitals in China and rarely reported in previous studies. Further multi-center survey was warranted to investigate the treatment status of aSAH.

## Conclusion

In this study, treatment delay was caused by pre-hospital delay including delayed admission and delayed transfer. Over 90% of patients could be treated at the same day of admission in our high-volume center. Younger age and good Fisher score were significantly related with admission delay. However, admission delay was further significantly correlated with better clinical outcome.

## Data Availability

The datasets involved during the current study are available from the corresponding author on reasonable request.

## Abbreviations

aSAH: Aneurysmal subarachnoid hemorrhage
AVM: Arteriovenous malformation
mRS: Modified Rankin Scale
SD: Standard deviation
PcomA: Posterior communicating artery
AcomA: Anterior communicating artery
MCA: Middle cerebral artery
ICA: Internal cerebral artery
PC: Posterior circulation
ACA: Anterior cerebral artery
